# Evaluation of ceftazidime/avibactam alone and in combination with amikacin, colistin and tigecycline against *Klebsiella pneumoniae* carbapenemase-producing *K*. *pneumoniae* by in vitro time-kill experiment

**DOI:** 10.1371/journal.pone.0258426

**Published:** 2021-10-14

**Authors:** Fangzhou Wang, Qian Zhou, Xiuwen Yang, Yan Bai, Junchang Cui

**Affiliations:** 1 Department of Respiratory Diseases, Chinese People’s Liberation Army General Hospital, Beijing, China; 2 Department of Respiratory Diseases, People’s Hospital of Hainan District, Wuhai, China; 3 Center for Life Sciences, School of Life Sciences, Yunnan University, Chenggong District, Kunming, China; Suez Canal University, EGYPT

## Abstract

*Klebsiella pneumoniae* carbapenemase-producing *K*. *pneumoniae* (KPC-Kp) poses a major threat to human health worldwide. Combination therapies of antibiotics with different mechanisms have been recommended in literatures. This study assessed *in vitro* antibacterial activities and synergistic activities of ceftazidime/avibactam alone and in combinations against KPC-Kp. In total, 70 isolates from 2 hospitals in Beijing were examined in our study. By using the agar dilution method and broth dilution method, we determined the minimum inhibitory concentration (MIC) of candidate antibiotics. Ceftazidime/avibactam demonstrated promising susceptibility against KPC-Kp (97.14%). Synergistic activities testing was achieved by checkerboard method and found ceftazidime/avibactam-amikacin displayed synergism in 90% isolates. Ceftazidime/avibactam-colistin displayed partial synergistic in 43% isolates, and ceftazidime/avibactam-tigecycline displayed indifference in 67% isolates. In time-kill assays, antibiotics at 1-fold MIC were mixed with bacteria at 1 × 10^5^ CFU/ml and Mueller-Hinton broth (MHB). Combinations of ceftazidime/avibactam with amikacin and tigecycline displayed better antibacterial effects than single drug. Ceftazidime/avibactam-colistin combination did not exhibit better effect than single drug. In KPC-Kp infections, susceptibility testing suggested that ceftazidime/avibactam may be considered as first-line choice. However, monotherapy is often inadequate in infection management. Thus, our study revealed that combination therapy including ceftazidime/avibactam colistin and ceftazidime/avibactam tigecycline may benefit than monotherapy in KPC-Kp treatment. Further pharmacokinetic/pharmacodynamic and mutant prevention concentration studies should be performed to optimize multidrug-regimens.

## Introduction

*Klebsiella pneumoniae* carbapenemase-producing *K*. *pneumoniae* (KPC-Kp) has spread widely and posed a heavy burden on the healthcare system and economy. According to a report from the China antimicrobial surveillance network (CHINET) [1], *Klebsiella pneumoniae* is the second most widespread pathogen among all Gram-negative bacteria. KPC-Kp has become one of the most important multidrug-resistant bacterial pathogens and KPC-Kp infection is a major risk to health of immunosuppressed and debilitated patients. Previous researches also reveal that the emergence of multidrug-resistant bacterial pathogens from different origins including humans, poultry, cattle, and fish that increase the need for routine application of the antimicrobial susceptibility testing to detect the antibiotic of choice as well as screening of the emerging MDR strains [2–4]. KPC-Kp mainly gains resistance to carbapenems and other antibiotics by the production of extended-spectrum beta-lactamases and K. pneumoniae carbapenemase enzymes [5–7] and the prevalence of KPC-Kp has resulted in failed treatment and high mortality. Antibiotics including carbapenems, colistin, tigecycline and aminoglycosides exhibit promising activity against *Klebsiella pneumoniae*. However, due to the extensive use, resistance to these antibiotics has developed in many regions.

Ceftazidime/avibactam is a combination of a third-generation cephalosporin and a novel non-beta-lactam beta-lactamase inhibitor. Ceftazidime/avibactam displays promising results against Gram-negative bacteria, including KPC-positive *Enterobacteriaceae* [8–10], and thus is considered as a valuable option for treatment. Avibactam is a novel beta-lactamase inhibitor that revives the activities of antibiotics against beta-lactamases producing pathogens, including Ambler class A, class C and a few class D beta-lactamases [11,12]. Despite natural resistant pathogens, such as *Acinetobacter baumannii*, isolates resistant to ceftazidime/avibactam have been reported in North America and some European countries, posing a threat to the last resort antibiotic options. One recent study reported the emergence of resistance under selection pressure with ceftazidime/avibactam alone [13]. This finding suggests that resistant strains may threaten the efficacy of ceftazidime/avibactam in the near future. A single substitution mutation of the KPC-2 gene and mutations that result in variant KPC-3 enzymes significantly increase the MIC of ceftazidime/avibactam. Given the wide use of ceftazidime/avibactam, these resistant mutant genes are likely to disseminate by horizontal gene transfer.

Previous studies demonstrate that susceptible strains exhibit an extreme low probability of developing mutations to survive in the presence of two drugs with different mechanisms. Therefore, multidrug regimens can reduce the chance of resistance emerging and combination therapy based on ceftazidime/avibactam should be considered. Some studies have investigated the interactions between ceftazidime/avibactam and other antibiotics [14]. However, few studies have investigated the *in vitro* interactions between ceftazidime/avibactam and other antibiotics against KPC-Kp. The objective of this study was to assess the ability of different combinations based on ceftazidime/avibactam to optimal treatment for KPC-Kp infections. In order to evaluate the *in vitro* synergistic activity of ceftazidime/avibactam alone and combined with colistin, amikacin and tigecycline respectively against KPC-Kp, we carried out our study against 70 KPC-Kp isolates with candidates mentioned above. Colistin is a bactericidal agent, damaging the external outer membrane of *Gram-negative bacilli* by impairing the LPS three-dimensional structure [15]. Tigecycline also inhibits the protein synthesis process of *Gram-negative bacilli* [16], and amikacin can be categorized as an aminoglycoside that can interfere the process of translation [17].

## Materials and methods

### Ethical statement

The experiment does not contain animal or human participants, and there is no need for the ethic approval.

### Bacteria isolates

A total of 70 non-duplicate isolates of KPC-producing *K*. *pneumoniae* were collected from patients with bloodstream infections in 2 hospitals in Beijing, China, from June 2014 to December 2016. All isolates were identified using the VITEK 2 Compact System (bioMérieux, Marcy-toilette, France).

### Identification of KPC-producing *K*. *pneumoniae*

MIC testing strictly followed the guidance of Clinical and Laboratory Standards Institute (CLSI) [18]. Agar dilution method was carried out to the MIC of carbapenems using Mueller Hinton agar (Difco, Franklin Lakes, NJ, USA) and meropenem (National Institute for the Control of Pharmaceutical and Biological Products, NICPBP, Beijing, China). In this test, *Escherichia coli* ATCC25922 was used as quality control strain. The results were examined as described in CLSI and revealed that all strains were resistant to carbapenems (MIC ≥8 mg/L).

### The bla_KPC_ gene detection

The bla_KPC_ gene was detected in all isolates mentioned above with a HiPure Bacterial DNA Extracting Kit. The PCR reaction was performed in a 20-μl volume containing 10μl 2 X PCR mix (Tianjing bio, Shanghai, China), 0.4μl of each primer and 1μl of DNA template. DDH_2_O was added to the volume to 20μl. The primers used are listed in S1 Table.

### Antimicrobial susceptibility testing

The MICs of ceftazidime, ceftazidime/avibactam, amikacin, colistin and tigecycline for 70 isolates were evaluated using the agar dilution method and broth dilution [18]. In this experiment, approximately 10^4^ Colony-Forming Unit (CFU) bacterial isolates were inoculated in the Mueller Hinton agar and Mueller-Hinton broth (Difco, Franklin Lakes, NJ, USA) plates that contains a series of 2-fold dilutions of each antibiotic. These plates were incubated at 37°C for 24 hours. MIC was defined as the lowest concentration that produced no visible growth of bacterial isolates. Breakpoints of ceftazidime/avibactam, amikacin and colistin were determined according to CLSI, and the breakpoint of tigecycline was determined according to FDA. Avibactam was purchased from Meilunbio (Meilunbio, Dalian, China). Amikacin, ceftazidime and colistin standards were purchased from NICPBP. Tigecycline was obtained from Wyeth Pharmaceutical (Wyeth Pharmaceutical, Philadelphia, PA, USA).

### Synergistic activity testing

Based on the results of susceptibility testing, a total of 30 KPC-Kp isolates that were susceptible to ceftazidime/avibactam, amikacin, colistin and tigecycline were randomly selected for synergistic testing. The checkerboard method was used in this experiment according to previous study [16], and all antibiotics were diluted in MHB to various concentrations. The bacterial suspension (1 × 10^5^ CFU/ml) was diluted and added to 96-wells plates. After been inoculated, mixtures were cultivated in 37°C for 20 hours. Fractional inhibitory concentration index (FICI) indicated interactions of drug combinations and was assessed by (MIC of drug A in combination/MIC of drug A alone) + (MIC of drug B in combination/MIC of drug B alone). FICI values were indicated the following: synergism, FICI≤0.5; partial synergism, 0.5<FICI <1; additivity, FICI = 1; indifference, 1<FICI <2; and antagonism, FICI >2.

### Time-kill assay

Eight isolates that were susceptible to ceftazidime/avibactam, colistin and tigecycline were randomly selected for this test. Among these isolates, 6 were susceptible to amikacin and 2 were resistant to amikacin. The Time-kill assay was performed according to previous study [16]. Before mixing with MHB, agents were diluted to 1-fold MIC. For isolates susceptible to ceftazidime/avibactam but resistant to amikacin, the concentration of amikacin was adjusted to the susceptibility breakpoint according to CLSI (16mg/L). Bacterial suspensions were adjusted to 1 × 10^5^ CFU/ml by MHB and mixed with agent dilutions. These mixtures were cultivated at 37°C for 24 hours, and samples were collected at 0, 3, 6, 12, 24 hours separately to count the number of colonies. To achieve this, specimens were 10-fold diluted serially if necessary, and time-kill curves were generated based on the counting results.

Bactericidal was considered when a reduction of ≥ 3 log10 CFU/ml. Compared with the most active drug in the pair, a further reduction of ≥ 2 log10 CFU/ml in combination was defined as synergism, a reduction of <2 log10 CFU/ml represented indifference, and an increment of ≥2 log10 CFU/ml was defined as antagonism [16].

## Results

Among 70 KPC-Kp isolates, 97.14% of isolates were susceptible to ceftazidime/avibactam and all isolates were resistant to ceftazidime alone. The rates of susceptibility to amikacin, tigecycline and colistin were 44.29%, 88.57% and 97.14%, respectively. The MIC_50_ and MIC_90_ of ceftazidime/avibactam were 1mg/L and 2mg/L, respectively. All results are shown in Table 1.

**Table 1:** *In vitro* activity of ceftazidime/avibactam, amikacin, colistin and tigecycline against *Klebsiella pneumoniae* carbapenemase-producing *K*. *pneumoniae*.

Antibiotics	MIC (mg/L)			Susceptibility (n/%)		
	MIC range	MIC_50_	MIC_90_	S	I	R
Ceftazidime	32->256	>256	>256	0	0	70(100%)
CEF/AVI	0.25–16	1	2	68(97.14%)	0	2(2.85%)
Amikacin	2–64	>64	>64	31(44.29%)	4(5.71%)	35(50%)
Colistin	0.25–8	0.25	0.5	68(97.14%)	0	2(2.85%)
Tigecycline	1–16	1	4	62(88.57%)	4(5.71%)	4(5.71%)

CEF/AVI = Ceftazidime/Avibactam, S = Susceptibility, I = Intermediate susceptibility, R = resistance.

**Fig 1:**
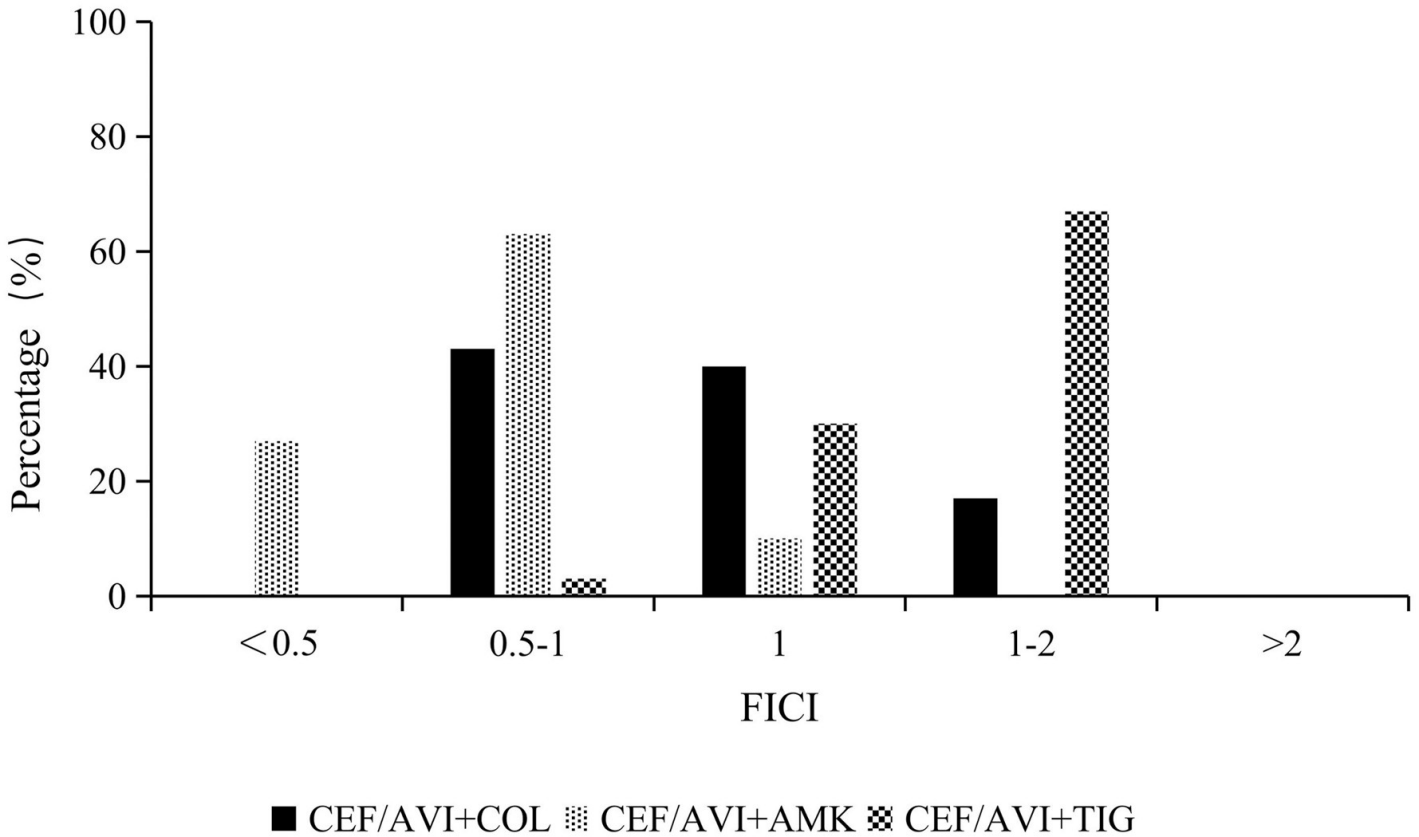
FICI distribution of ceftazidime/avibactam combined with amikacin, colistin and tigecycline (n = 30).

The synergistic activity results for ceftazidime/avibactam susceptible isolates revealed that the ceftazidime/avibactam and amikacin combination displayed synergistic and partial synergistic activity in 90% isolates. Additivity was noted for the remaining isolates. Partial synergism between ceftazidime/avibactam and colistin was found in 43% isolates, and this combination exhibited additivity in 40% isolates. Regarding the ceftazidime/avibactam and tigecycline combination, partial synergism, additivity and indifference were noted in 3%, 30% and 67% of isolates, respectively. Fig 1 shows the FICIs of synergistic activity testing.

**Fig 2:**
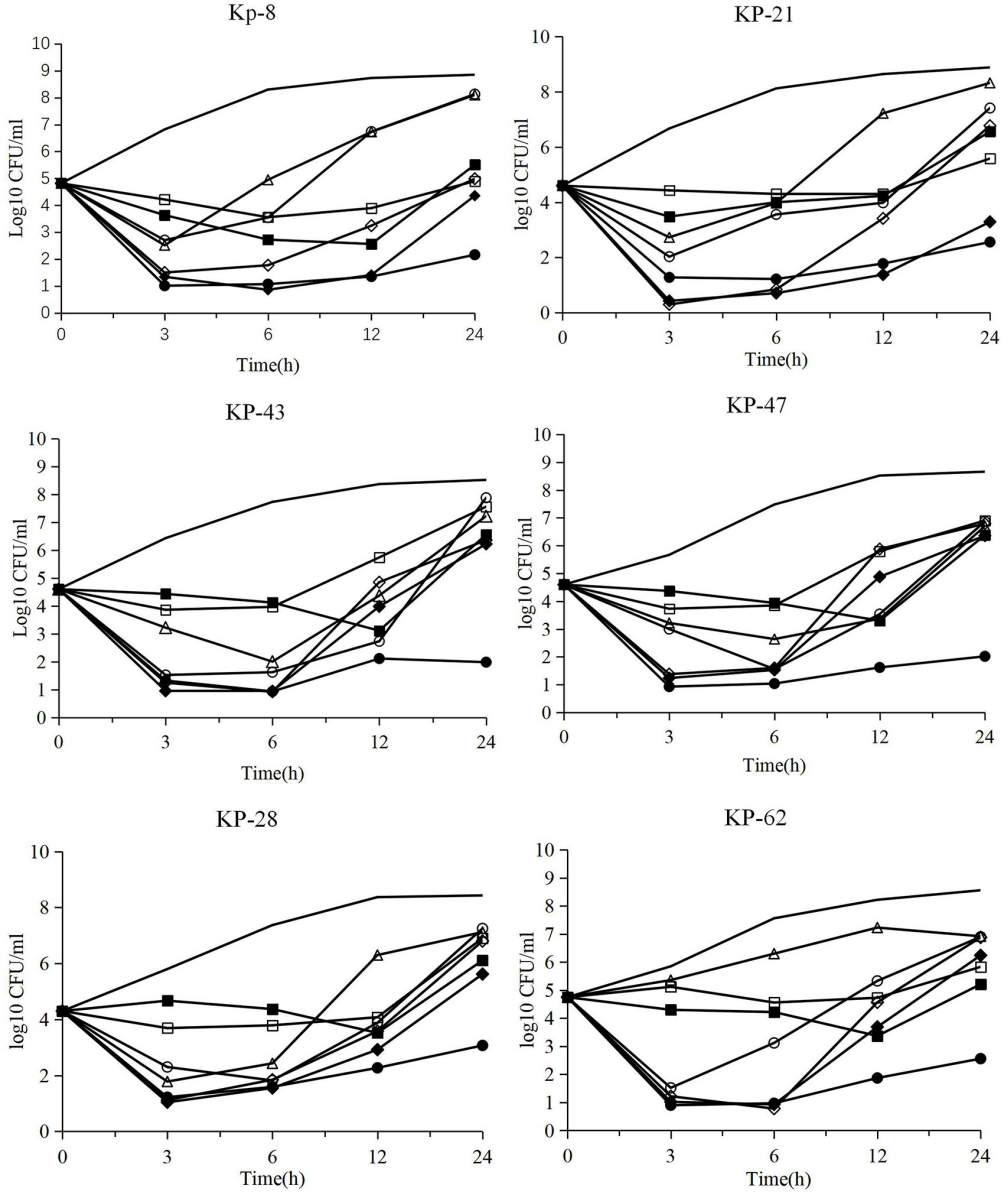
In vitro time kill curves of ceftazidime/avibactam (CEF/AVI), amikacin (AMK), colistin (COL) and tigecycline (TGC) alone and in combinations against KPC-*K*. *pneumoniae* isolates susceptible to ceftazidime/avibactam, amikacin, colistin and tigecycline. Line for blank control, CEF/AVI, ○AMK, ◇COL, □TGC, ●CEF/AVI+AMK, ◆CEF/AVI+COL, ■CEF/AVI+TGC.

**Fig 3:**
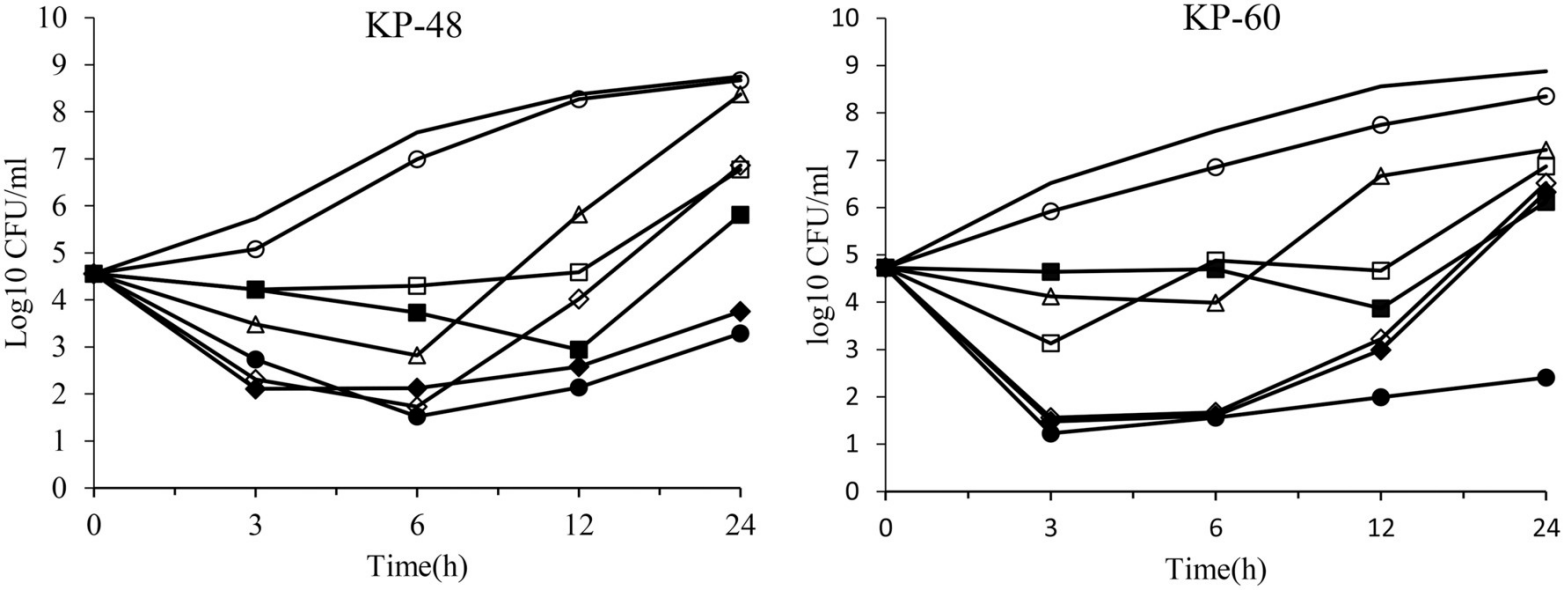
In vitro time kill curves of ceftazidime/avibactam (CEF/AVI), amikacin (AMK), colistin (COL) and tigecycline (TGC) alone and in combinations against KPC-*K*. *pneumoniae* (KP48 and KP60 were susceptible to ceftazidime/avibactam, colistin and tigecycline but resistant to amikacin). Line for blank control, △CEF/AVI, ○AMK, ◇COL, □TGC, ●CEF/AVI+AMK, ◆CEF/AVI+COL, ■CEF/AVI+TGC.

The time kill assays results are shown in Figs 2 and 3. For six isolates that were susceptible to ceftazidime/avibactam, amikacin, colistin and tigecycline, tigecycline exhibited bacteriostatic activity, whereas ceftazidime/avibactam, amikacin and colistin alone exhibited bactericidal activity in the first 6 hours. However, bacteria started to regrow after 6 hours. Ceftazidime/avibactam-amikacin displayed better antimicrobial activity than monotherapy. The addition of colistin to ceftazidime/avibactam did not obviously improve antimicrobial activity. Exponential regrowth of bacteria was observed after an incipient decrease in isolates. For amikacin resistant strains, ceftazidime/avibactam enhanced the activity of amikacin and significantly reduced the number of bacterial colonies. Synergistic activity and bactericidal activity were observed in ceftazidime/avibactam-amikacin combinations.

## Discussion

In susceptibility testing, avibactam restored the antimicrobial activity of ceftazidime which is consistent with other study that the addition of avibactam to ceftazidime resulted in 4-fold to 512-fold reductions in MIC of beta-lactamase-producing isolates [19]. Ceftazidime/avibactam demonstrated promising *in vitro* activity against clinical isolated strains of KPC-Kp. Based on the high susceptibility rates, ceftazidime/avibactam is considered as first-line choice for KPC-Kp. Despite the excellent activity, several studies have reported the emergence of avibactam-resistant isolates. *Klebsiella pneumoniae* isolates acquire resistance via beta-lactamase related amino acid substitutions, porin deficiencies and efflux pumps [20–22]. Also, the presence of high level of beta-lactamase expression may contribute to the ceftazidime/avibactam resistance. *K*. *pneumoniae* isolates carrying bla_KPC_ gene can impair the activity of ceftazidime/avibactam [23]. In a clinical trial, the median time of resistance development was 15 days, and the resistance was observed in as few as 10 days [22]. Therefore, single use of ceftazidime/avibactam may result in rapid enrichment of resistant isolates and result in treatment failure. In synergism testing, ceftazidime/avibactam-amikacin combination demonstrated the highest in vitro synergism against KPC-Kp. Ceftazidime/avibactam-colistin combination also displayed promising synergism against KPC-Kp. However, several studies have shown that ceftazidime/avibactam-colistin may not exhibit better efficacy *in vitro* and *in vivo* and should be used carefully.

In the time-kill assay, *in vitro* activity of ceftazidime/avibactam and other candidates alone and in combinations were tested against ceftazidime/avibactam susceptible KPC-Kp strains. According to the results of single drug time-kill testing, ceftazidime/avibactam alone failed to suppress the regrowth against susceptible isolates. Since single-drug therapy is often inadequate, combination therapy has been recommended to prevent resistance. In combination time-kill testing, combination therapy demonstrated better *in vitro* activities against KPC-Kp. Notably, ceftazidime/avibactam amikacin displayed the most effective activity against all six KPC-Kp strains. Our results were in agreement with previous studies that revealed the synergism between ceftazidime/avibactam and amikacin [14,24,25]. Due to the low susceptibility rate of amikacin among KPC-Kp in China, KPC-Kp isolates resistant to amikacin were examined in time-kill testing. Ceftazidime/avibactam-amikacin combination exhibited better activity than single drug and restored amikacin susceptibility. The potential mechanistic explanations for the synergism between ceftazidime/avibactam and amikacin are still unclear.

Gram negative bacteria gain resistance to ceftazidime/avibactam via mechanisms, including enzymatic resistance, modification of the antibiotic targets and efflux pumps [21]. Aminoglycosides reduce the activity of drug efflux pumps, therefore reducing resistance of other drugs and leading to collateral sensitivity [26,27]. However, efflux pumps are not the main mechanism of resistance to ceftazidime/avibactam [28]. Mutations in class A beta-lactamase that causes resistance in KPC-Kp have been widely reported. Amino acid substitutions in beta-lactamases caused by mutations contribute to the significant increase in MIC values. Aminoglycosides may disrupt protein synthesis and the membranes permeability of bacteria, enhancing bactericidal effects of the ceftazidime/avibactam and amikacin combination and demonstrating it potential to prevent resistance. The synergism of the ceftazidime/avibactam amikacin and tigecycline combinations indicates potential roles in preventing resistance. The probability that bacteria develop 2-steps mutations simultaneously to gain resistance to combination therapy is very low (<10^−10^). In addition, bacteria may develop resistance to one drug, which can lead to increased sensitivity to another drug with a different mechanism of action.

It is also notable that ceftazidime/avibactam-colistin combination did not exhibit better activity against KPC-Kp in time-kill testing. This result was consistent with previous studies. Borjan suggested that the ceftazidime/avibactam-polymyxin B combination should be avoid given the potential for toxicity and the lack of synergism [29]. A study in North America found that colistin failed to potentiate ceftazidime/avibactam in the eradication of carbapenem-resistant *Enterobacteriaceae* and suppress the emergence of ceftazidime/avibactam resistance [30]. An *in vivo Galleria mellonella* survival model analysis described by Borjan showed that ceftazidime/avibactam-polymyxin B did not improve *in vivo* survival. Therefore, given the renal toxicity of colistin, ceftazidime/avibactam-colistin should be used carefully.

There are several limitations in our study. The *in vitro* activities and synergistic activities reported here did not represent clinical effects. *In vitro* pharmacokinetic/pharmacodynamics (PK/PD) experiments and *in vivo* experiments, which provide more details on the effects of antibiotics combinations, should be performed. The effects of the immune system are also important as it may interact with antibiotics [31,32]. In addition, to prevent the emergence of resistance, further studies that focus on mutant selection window (MSW) and mutant prevention concentration (MPC), which evaluate ability of drug combinations to prevent resistance, are needed [33]. Therefore, promising combinations can be further evaluated with *in vivo* PK/PD studies and MPC experiments before clinical use.

## Conclusion

Our study suggests that ceftazidime/avibactam is be considered as first-line choice. Compared with single drugs, ceftazidime/avibactam-amikacin combination, may exert synergistic effects and prevent the emergence of ceftazidime/avibactam resistance. This combination could be an effective therapy for KPC-Kp infections in clinical practice.

## Supporting information

S1 Fig PEGE results for isolates of carbapenem-resistant *K*. *pneumoniae* with blaKPC gene. Plasmid bands are shown as linearized fragment on the gel. DS5000 was used as marker, from the top to the bottom, it is 5000, 3000, 2000, 1500, 1000, 750, 500, 250, 100kb.
(TIF)

S1 Table PCR testing primer and cycling conditions for the detection of blaKPC gene of KPC-Kp. (DOCX)

S1 Data (XLS)
