# Supplementary figures and images for: Evaluation of ceftazidime/avibactam alone and in combination with amikacin, colistin and tigecycline against Klebsiella pneumoniae carbapenemase-producing K. pneumoniae by in vitro time-kill experiment
Source: PLoS One. 2021 Oct 14;16(10):e0258426. doi: 10.1371/journal.pone.0258426 (PMC8516195; doi:10.1371/journal.pone.0258426)

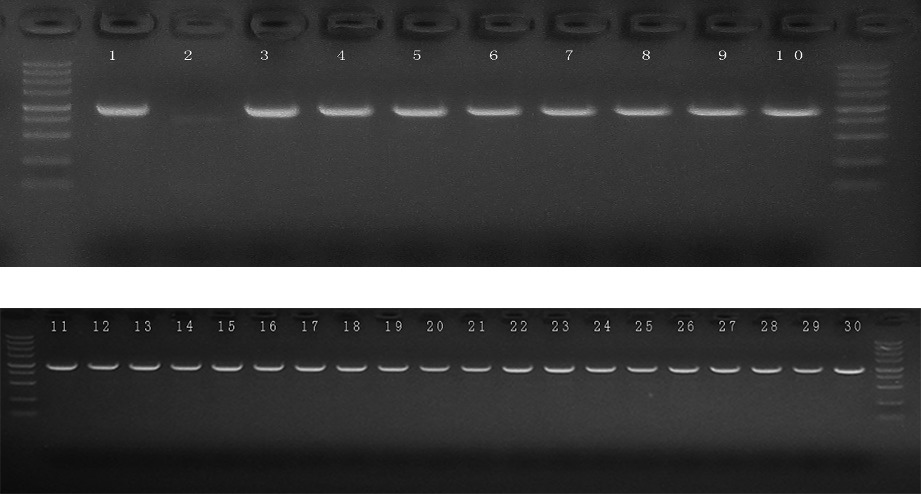

Supplement: S1 Fig — Plasmid bands are shown as linearized fragment on the gel. DS5000 was used as marker, from the top to the bottom, it is 5000, 3000, 2000, 1500, 1000, 750, 500, 250, 100kb. (TIF) [file pone.0258426.s001.tif]
