# Supplementary material for: Evaluation of ceftazidime/avibactam alone and in combination with amikacin, colistin and tigecycline against Klebsiella pneumoniae carbapenemase-producing K. pneumoniae by in vitro time-kill experiment
Source: PLoS One. 2021 Oct 14;16(10):e0258426. doi: 10.1371/journal.pone.0258426 (PMC8516195; doi:10.1371/journal.pone.0258426)
Supplement: S1 Table — (DOCX) [file pone.0258426.s002.docx]

**PCR testing primer and cycling conditions for the detection of bla_KPC_ gene of KPC-Kp.**

| Target gene | Primer 5`-3` | Size(bp) | PCR cycling conditions  (35 cycles) | | | Reference |
| --- | --- | --- | --- | --- | --- | --- |
| bla_KPC-2_ | TGTCACTGTATCGCCGTC | 900 | 94℃  1min | 52℃  1min | 72℃  1min | [1] |
|  | CTCAGTGCTCTACAGAAAACC |  |  |  |  |  |

Reference

1. Bratu S, Tolaney P, Karumudi U,et al. Carbapenemase-producing Klebsiella pneumoniae in Brooklyn, NY: molecular epidemiology and in vitro activity of polymyxin B and other agents. J Antimicrob Chemother. 2005 Jul;56(1):128-32. doi: 10.1093/jac/dki175.
